# Toxicity and health effects of delta-8, delta-9, and delta-10-tetrahydrocannabinol and unregulated cannabinoids in vaping products

**DOI:** 10.1016/j.toxrep.2026.102202

**Published:** 2026-01-08

**Authors:** Karen Lin, Yehao Sun, Rhea Raghu, Parth Suharu, Felix Effah, Irfan Rahman

**Affiliations:** Department of Environmental Medicine, University of Rochester Medical Center, Rochester, NY, United States

**Keywords:** Cannabis, CBD, THC, EVALI, Vaping, Lungs

## Abstract

Hemp-derived cannabinoids (CBDs) such as Δ8- and Δ10-tetrahydrocannabinol (THC) in cannabis e-cigarettes have been growing in popularity among youth, causing great concern for their health effects. Previous novel lung injury outbreaks, such as E-cigarette or Vaping Use-Associated Lung Injury (EVALI), were associated with the rising use of e-cigarettes and vaping products. Toxicological studies have revealed that chronic exposure to cannabis vapor can cause adverse brain and pulmonary effects. Hemp products are classified as cannabis and set a limit of no more than 0.3 % Δ9-THC, while products containing more than 0.3 % are defined as ‘marijuana.’ This has led to the proliferation of hemp-derived intoxicating cannabinoids, such as Δ8- and Δ10-THC, in addition to cannabidiol (CBD), cannabinol (CBN), cannabigerol (CBG), and Δ9-THC appearing in combination products. CBD frequently serves as a significant component of hemp-derived formulations, making it a central consideration for toxicological and regulatory evaluation as well. This phenomenon poses significant health risks to youth because these newer THC isomers and products are currently unregulated and not well-researched, yet they are still widely available. Therefore, we have examined the pharmacology, toxicity, potential therapeutic uses and possible health risks of several THC and hemp-derived cannabinoids. This review draws insightful highlights to the public health consequences of secondary exposures to CBD and THC, and their molecular mechanisms of action. It underscores the urgency for a regulatory oversight over unregulated cannabinoid markets to prevent toxicity of vaping-related health crises and other rapidly emerging cannabis health disorders, like the cannabinoid hyperemesis syndrome (CHS).

## Highlights

• Hemp-derived cannabinoids (Δ8-, Δ9, Δ10-, CBD) contribute to emerging toxicological health risks.
• Cannabis vaping is associated with pulmonary toxicity such as EVALI and CHS.
• THC and CBD both exert effects via CB1/CB2 receptors and NF-κB pathways.
• There is an urgent need for regulations based on toxicity of cannabinoids.

## Graphical Abstract

**Figure:**
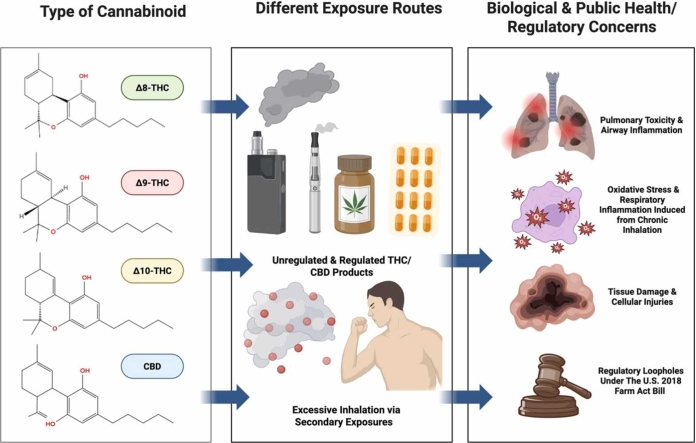

## 1 Introduction

In recent years, as hemp-derived cannabinoids (CBDs) are legal in most states, cannabis vaping has surged in popularity amongst many young adults and teenagers. Hence, there has been prominent attention surrounding the purity and unlabeled additives in cannabis e-cigarettes (CECs) that can pose a threat to health [1]. In adolescents specifically, the prevalence of lifetime CEC usage has doubled from 6.1 % in 2013 to 2016–13.6 % in 2019–2020 [2]. The increase in usage has mainly been due to the growing perception of cannabis being less harmful than traditional smoking methods, drawn from extensive reports highlighting the potential therapeutic efficacies for medical purposes [3]. However, CECs cause great concern to medical communities as they have previously been associated with an inflammatory lung disease, namely, E-cigarette or Vaping Use-Associated Lung Injury (EVALI) [3]. According to the Centers for Disease Control (CDC), as of February 2020, there have been a total of 2668 hospitalizations and deaths throughout the U.S. associated with e-cigarettes and EVALI [4].

In addition to respiratory issues like EVALI, habitual cannabis usage, especially through unregulated and high-potency cannabis products, has been associated with the cannabinoid hyperemesis syndrome (CHS) [5]. Symptoms of CHS, often characterized by episodes of abdominal pain, nausea, and vomiting can easily be overlooked [6], due to overlapping symptoms with other common diseases. In one report, an increase of 134–175 % was expected for annual prevalence rates of CHS [7]. The rise of this syndrome is also suspected to be closely linked with the proliferation of cannabis products amongst young adults, and the rapid emergence of newer unregulated THC products, synthetic cannabinoids, and newer THC isomers [8], amid regulatory gaps. Although most existing CHS cases involve chronic cannabis use with unclear and poorly documented routes of administration, this review specifically considers how the proliferation of high potency vaping products and hemp-derived cannabinoids may influence risk of CHS. Current systematic literature and empirical studies show some promising treatments for CHS [9], but scientific evidence for pharmacologic treatment is still limited, underscoring the importance of awareness of health risks associated with cannabinoid consumption and the need for more clinical trials to define an optimal treatment for this emerging syndrome.

Generally, CECs contain three primary chemical constituents, including tetrahydrocannabinol (THC), medium-chain triglycerides (MCT), and vitamin E acetate [10]. Although many recent studies have identified vitamin E acetate as the primary potential causative agent in CECs, other unlabeled aerosol components and direct effects of aerosolized THC cannot be unnoticed. There have also been reports before 2019 suggesting that many lung injuries continue to stem from cannabis inhalation, indicating that other unlabeled cannabis components or additives in CECs may be responsible [3]. Many CECs associated with lung injuries like EVALI have also been found to contain unnatural cannabinoid distributions and synthetic forms of vitamin E acetate, often added to THC containing e-liquids as a cutting agent [1].

In studies involving *in vitro* mouse cells, when examining the effects of vitamin E acetate, MCT, and CBD/counterfeit vape cartilages on cytotoxicity and inflammatory responses, it was observed that increasing exposure to these constituents elicits a differential inflammatory response in both epithelial cells and monocytes [10]. Specifically, it was found that infiltration of neutrophils and lymphocytes correlates with increased levels of exotoxin, interleukin-6 (IL-6), and granulocyte colony-stimulating factor (G-CSF) in the bronchoalveolar lavage fluid (BALF), which are all well-known inflammatory markers implicated in lung inflammation. Notably, exposing cells to CBD or counterfeit vape aerosols had a more toxic effect than exposure to vitamin E acetate and MCT [10]. These findings highlight how THC vaping products can pose serious health risks, which can be further exacerbated by regulatory loopholes and inaccurate commercial labeling.

Current federal regulations surrounding the US 2018 Farm Act Bill contain some loopholes, theoretically allowing vaping manufacturers to sell hemp-derived cannabinoids other than Δ9-THC and for the capitalization and marketing of Δ8-THC products [3]. In fact, as of May 2023, recent data show that most common product types across retail websites are 43 % disposable vapes, 29 % edibles, 18 % cartridges, 7 pre-rolls, 2 % flower, 1 % dabs, and < 1 % vape pods [11]. In addition, recent data also identified 26 toxic compounds in the products, with Δ8-THC, Δ9-THC, Δ10-THC, Δ11-THC, HHC, and others being the most common [11]. Worse yet, in another study analyzing 27 products from 10 brands using NMR, GC-MS, and ICP-MS, none were found to have accurate labeling, with 11 products containing unlabeled cutting agents [1]. Side products of these cannabis vapes also include heavy metals, olivetol, Δ4-iso-tetrahydrocannabinol, and 9-ethoxyhexahydrocannabional [1]. Many cannabis vape users, particularly young adults, may be at potential risk for adverse health effects from inhaling high quantities of unknown additives [12], [13]. As the cannabinoid market continues to expand, there will be even more synthetic cannabinoids beyond the commonly known Δ8-THC [14]. On December 18, 2025, the White House announced further research on THC products, and reclassified marijuana from a Schedule I to a Schedule III controlled substance.

Furthermore, other novel cannabis vaporizer ingredients such as Δ10-THC and hexahydrocannabinol, also known as “legal highs,” continue to emerge on the market, causing great concern for medical communities [15]. Yet, their toxicology and pharmacology still have not been fully researched. This underscores the urgent need for further research on unlabeled additives and stricter regulatory oversight of unregulated vaping manufacturers to prevent another vaping-related health crisis. In this review, we analyzed hemp-derived cannabinoids and different types of tetrahydrocannabinols, as well as their pharmacology, toxicology and potential health benefits, therapeutic benefits, and risks. This review also aims to highlight the public health consequences of secondary exposures to CBD and THC while discussing the urgency to close the current loophole in federal regulations.

## 2 Methods

A comprehensive literature review was conducted through freely accessible papers across PubMed and Google Scholar from 2014 to 2025 using the following keywords: “cannabis,” “cannabidiol,” “tetrahydrocannabinol,” “hemp,” “toxicity,” “delta 8-THC,” “delta 9-THC,” “delta 10-THC,” “cannabis hyperemesis syndrome,” “inflammation,” “THC isomers,” “THC pharmacology,” “secondary exposure,” “NF-kappaB,” and “biotoxicity.” Inclusion criteria comprised of peer-reviewed articles, federal regulatory documents, and scientific studies relevant to cannabinoid toxicology and regulation. Given the limited regulatory attention to cannabinoid analogs in recent years, there are limited regulatory publications regarding this focus, and hence selected cited literature and federal regulatory documents published prior to 2014 were included when directly relevant. Each abstract, methods, and discussion were screened for relevance, followed by a full-text review. Evaluation of each database involved the type of study design (e.g. *in vitro* vs. *in vivo* experiments), sufficiency of sample size, and clarity of reporting. Literature evidence were then compiled and synthesized to highlight current understanding surrounding toxicants in E-cigarettes; the toxicology and chemistry of different cannabinoids; pharmacology gaps THC isomers; present loopholes in cannabinoid FDA frameworks; harmful and beneficial effects of CBD/THC; cannabinoid hyperemesis syndrome’s link to THC use and its underlying biological mechanism; biomarkers of CBD/THC toxicity; current public health concern on CBD/THC use and secondary exposures; further regulatory recommendations; and present policies surrounding cannabis and cannabis research.

## 3 Cannabinoids (CBD) versus tetrahydrocannabinol (THC): overview

CBD and THC are the two major classes of phytocannabinoids isolated from cannabis plants, differing in their psychoactive effects and receptor interactions [3]. Currently, there are over 100 phytocannabinoids and over 500 chemical compounds derived from cannabis plants [16]. Presently, CBD and Δ9-THC are the most researched phytocannabinoids [17]. The endocannabinoid system, critical for homeostasis and neuroplasticity, consists of two main receptors: cannabinoid 1 receptor (CB1) and cannabinoid 2 receptor (CB2), which are primarily responsible for the physiological and psychoactive effects of cannabis [18]. CB1 is located on multiple neurons in the central nervous system. At the same time, CB2 is primarily expressed in glial cells and the enteric nervous system, while CB1 typically show higher expression than CB2 in human lungs [3], [18]. THC produces more psychotomimetic effects by activating CB1 and can have other immunologic effects by activating CB2 [18]. Specifically, the administration of Δ9-THC is most potent compared to other forms of THC like Δ8- and Δ10-, producing the feeling of ‘high,’ perceptual alterations, delusions, cognitive memory deficits, and verbal complications at higher doses [16], [18]. This is the case due to Δ9-THC’s role as a partial agonist at both CB1 and CB2 receptors and its strong affinity compared to other phytocannabinoids [19]. Similarly, Δ8-THC acts as a partial agonist at CB1 receptors, but with a weaker affinity and efficacy [20]. Unlike Δ9- and Δ8- THC, Δ10-THC trans- and cis- isomers are generally less researched, but *in vitro* studies have displayed its function as a CB1 antagonist [21].

On the other hand, CBD is often well tolerated, with few to no psychotropic effects. Despite CBD’s low affinity for CB1 and CB2 receptors, it is readily capable of opposing THC’s activation of these receptors when both are present [22]. CBD is non-intoxicating and often described clinically as non-psychotropic, influencing many physiological processes [18]. Yet, CBD can still exert central nervous system effects, including antiepileptic and anxiolytic actions [18]. Indeed, recent reports have noted that CBD may have potential clinical benefits for alleviating anxiety, cancers, epilepsy, and neuropathic pain [16], [23].

However, recent studies have also examined that when CBD and THC are combined in pharmaceutical preparations or clinical uses in a 1:1 ratio, CBD acts as an antagonist to psychoactive outcomes and counteracts adverse effects from high doses of THC without interfering with relaxation effects [16], [24]. There have also been contradictory studies arising from differences in dose ratios, analyses, and administration methods, leading to several discrepancies [25]. Nonetheless, research shows that CBD decreases the psychotomimetic effects of cannabis by lowering the THC effects [18]. Despite potential therapeutic applications, loopholes in current federal regulations cannot be unseen.

### 3.1 Detailed pharmacology of THC isomers

As discussed previously, THC can also exist in multiple isomeric forms, including Δ9- (the primary psychoactive component), Δ8-, and Δ10-THC. Beyond these, several *cis-* and *trans-*stereoisomers also exist, each with distinct pharmacological properties. Still, there are some considerable gaps and limited understanding of THC isomers, particularly in its potency, toxicity profile, and metabolism [26]. It is well known that while Δ9-THC serves as a strong agonist at the CB1 cannabinoid receptor, Δ8-THC and Δ10-THC exhibit a weaker affinity for CB1, which accounts for their reduced psychoactivity [21]. In fact, studies have highlighted that emerging Δ10-THC isomers (specifically *trans-, cis-,* HHC, and others) may even serve as an antagonist for CB1 [21] (Table 1, Table 2). Additionally, recent findings indicate that Δ9-THC may also interact with other receptors, including the GR alpha (GRα), either directly or indirectly, to override the effect of steroids via CB1/CB2-GRα interaction, thereby rendering the steroids ineffective in human macrophages [27]. Furthermore, CBD can differentially regulate basal pro-inflammatory response and attenuate both lipopolysaccharide (LPS)-induced cytokine release and NF-κB activity in monocytes, similar to dexamethasone [27]. However, the full spectrum of receptor binding profiles and functional activities for many isomers, particularly those produced synthetically, remains uncharacterized, unclear, and unstudied in humans.

**Table 1:** Major phytocannabinoids and their cannabinoid receptor activity/psychoactivity.

**Cannabinoid**	**CB1 Activity**	**CB2 Activity**	**Relative Affinity**	**Psychoactivity**
**Δ9-THC**	Partial agonist [19].	Partial agonist [19].	Strong	Main psychotropic constituent of cannabis [19]. Most potent compared to Δ8- and Δ9-THC [16], [18].
**Δ8-THC**	Partial agonist [20].	Activates CB2, but with a lower potency than Δ9-THC [20].	Moderate	Moderate/ Milder psychotropic profile than Δ9-THC [20].
**Δ10-THC** **(*****cis-*****&*****trans-*****)**	Antagonist (*in vitro)*[21]. Less potent at CB1 receptors [21].	Uncertain and weak activity at CB2 [21].	Limited data	Unlikely to have THC-like psychoactivity [21].
**CBD**	Antagonist & Negative allosteric modulator [22].	Antagonist [22].	Low direct affinity	Few to no psychotropic effects at therapeutic doses [22].

**Table 2:** Chemical analog, molecular formula, characteristics, and structure of Δ8-THC, Δ9-THC, and Δ10-THC.

**THC**	**Chemical Properties/ Origin**	**Characteristics**	**Structure**
**Δ8-THC**	Molecular formula: $C_{21}H_{30}O_{2}$[28] Molecular Weight: 314.5 g/mol [28] While Δ8-THC is naturally present in only trace amounts in cannabis plants, nearly all commercial Δ8-THC is often artificially synthesized from CBD via acid-catalyzed isomerization [29].	Δ8-THC is more stable than Δ9-THC due double bond positioning on the 8th carbon, causing lower reactivity [30]. Less binding to CB1 receptors compared to Δ9-, as such causes less psychoactive effects [30].	
**Δ9-THC**	Molecular Formula: $C_{21}H_{30}O_{2}$[31] Molecular Weight: 314.5 g/mol [31] Naturally abundant and directly isolated from cannabis plants [32].	Less stable under light and heat due to double bond being on ninth carbon. Has a higher reactivity with electrophiles and oxidation agents leading to a faster degradation over time [30].	
**Δ10-THC** **(*****trans- and cis-*****)**	Molecular Formula: $C_{21}H_{30}O_{2}$[33], [34] Molecular Weight: 314.5 g/mol [33], [34] Δ10-THC isomers are primarily created synthetically or semi-synthetically via isomerization from hemp-derived CBD [21].	Potentially similar stability as Δ8-THC Limited data on potency and psychoactive effects	***cis*****-Δ-10-THC (S configuration)*****trans*****-Δ-10-THC (R configuration)**

Another interesting finding regarding Δ9-THC isomers reported in Schafroth et al.’s study highlighted that (−)-Δ9-*cis*-THC acts as a partial agonist with lower binding affinities to cannabinoid receptors compared to the more prevalent (−)-Δ9-*trans*-THC [35]. On the other hand, (+)-Δ9-*cis*-THC enantiomer appeared largely inactive at cannabinoid receptors. However, it showed evidence of selective inhibition of different endocannabinoid-degrading enzymes [35], which can indicate potential pharmacological activities and an inability to produce THC-like effects. This finding may introduce a further nuanced pharmacological profile of (+)-Δ9-*cis*-THC and its potential as a scaffold for novel therapeutics targeting the endocannabinoid system beyond the classical receptor-mediated effects if further research is conducted.

Furthermore, there is also a limited understanding of the toxicity and safety profiles of different THC isomers. For instance, Δ8-THC can be readily derived synthetically from CBD via acid-catalyzed reactions [29]. However, during this chemical process, various reaction conditions, such as temperature changes, reaction duration, type of solvent, and atmospheric exposure, can affect the overall yield of Δ8-THC, resulting in a mixture of toxic byproducts [36]. In addition to Δ8-THC, the catalytic process can also generate other non-natural THC isomers, including Δ7-THC, Δ10-THC, and Δ11-THC [36]. Nevertheless, many of these non-natural compounds have not been thoroughly evaluated for their pharmacological or toxicological effects on humans. This raises significant concern about the safety of commercially accessible Δ8-THC products on the market produced via the synthetic route. Hence, the pharmacological uncertainty of THC further underscores the need for a stricter regulatory oversight and toxicological studies on byproducts in Δ8-THC products.

Above all, analytical challenges are a further complication as the metabolites of many isomers are difficult to distinguish through traditional standard laboratory techniques. Because THC isomers have nearly identical structures, conventional laboratory methods such as chromatography and mass spectrometry remain a challenge when accurately quantifying different isomer metabolites since similar isomers tend to overlap in retention times and result in similar mass spectra [37]. However, recent advances in analytical chemistry, such as cyclic ion mobility spectrometry-mass spectrometry (IMS-MS) combined with silver ions, help amplify the subtle structural differences between different isomers by observing unique ways cannabinoids interact with silver ion particles [37]. Despite these advances, growing diversity in cannabinoid isomer structures would likely outpace current testing capabilities. Thus, the need for specific and accurate analytical methods is essential, as synthetic isomers of THC appear on the market.

## 4 Toxicants & toxicological concerns in e-cigarettes containing THCs

While it is well known that cannabinoids in e-cigarettes pose potential health risks, especially from their different pharmacological and psychoactive effects, counterfeit CECs consisting of electronic parts that are often unlawfully replicated or altered from original manufacturers can also cause significant harm due to the presence of various unknown chemical toxicants. Apart from vitamin E acetate being well-known as the primary causative agent in CECs linked to lung injuries, other potential toxicants such as metals, pesticides/plasticizers, solvent-derived hydrocarbons, silicon-linked compounds, and other terpenes in counterfeit cartridges have also been previously found to play a role in lung injuries [38]. Interestingly, these toxicants are primarily found in counterfeit cartridges because cutting agents such as seized drugs like butane hash oil, legal substances like MCT oil, and vitamin E acetate are used for production. All these chemicals induce oxidative stress and several lung inflammatory responses [39]. Several solvents like n-butane and dimethoxyethane, have been detected in both the liquid and vapor phases of counterfeit CECs [38]. The butane derivative is likely due to the cartridge production's “dabbing” extraction process. Case reports have revealed that when butane hash oil is heated at high temperatures, harmful derivatives and byproducts like methacrolein and benzene are produced, which are also pulmonary irritants and carcinogens [40].

Other compounds, such as undecane and decane hydrocarbons, commonly found in CECs, can cause respiratory tract irritation and central nervous system depression. If hydrocarbons are aspirated into the lungs, induced chemical pneumonitis may cause lesions and damage to alveolar and capillary membranes [38]. Significant levels of silica and tetramethyl silicates were also found in counterfeit cartridges. High levels of silico compounds inhaled can cause acute lung damage including pulmonary edema and lesions, through the formation of toxic secondary products from silicon dioxide and methanol [38]. Even worse, additional compounds found in e-cigarette counterfeit cartridges, including isoprene, toluene, acrolein, benzene, acetaldehyde, and ethylbenzene, are identified as harmful constituents by the FDA [38]. These chemical constituents, when inhaled, can react to form peroxides and self-polymerize upon exposure to oxygen, which can further catalyze the formation of other secondary harmful byproducts. Collectively, the presence of these harmful constituents in counterfeit CEC cartridges can emphasize their strong toxic role in severe respiratory injuries and chemical-induced pneumonitis, as evidenced in cases of EVALI.

### 4.1 Toxicology and chemistry of different cannabinoid structures

Beyond the non-cannabinoid toxicants previously discussed above, the intrinsic chemistry and toxicology of individual cannabinoid structures, Δ8-, 9-, and 10-THC, are also critical determinants of health. The three positional isomers, Δ8-, 9-, and 10-THC, all interact with the endocannabinoid system in humans with notable psychoactive effects [41]. However, they vary in potency and toxicological effects. Δ8-THC has its double bond at the 8th carbon. This structural change reduces the potency and is associated with dizziness, confusion, sedation, and anxiety [41], [42]. Animal studies have also brought up more toxicological concerns. For example, when Δ8-THC was administered to rats, dose-dependent reductions in body weight and relative organ weights were observed, including epididymis, heart, liver, lung, and spleen [43]. These findings may prove that exposure to high doses of Δ8-THC may interfere with metabolic processes and/or organ functions [43].

Unlike Δ8-THC, Δ9-THC is biosynthetically produced and is identified as the main potent psychoactive component of the Cannabis sativa plant [44]. Although the toxicity of Δ9-THC can generally cause side effects such as dizziness and cognitive impairments, serious adverse events are claimed to be typically rare and reversible [45]. In one recent study, it was found that Δ9-THC can significantly alter a brain’s dopamine levels by stimulating mesolimbic dopamine neurons. In turn, this can lead to elevated striatal dopamine, linked to neurological interference and addictive potential [44]. Preclinical studies have also shown that exposure to (−)-Δ9-THC during pregnancy can also lead to fetal toxicity and potential neurodevelopmental deficits [46]. Similarly observed, other studies have found that when prenatal maternal immune activation and Δ9-THC exposure co-occur, they can potentially lead to long-lasting neuroanatomical and behavioral changes in adulthood [47]. Specifically, in Guma et al.’s study, when researchers performed behavioral tests and utilized longitudinal MRI to observe discrepancies in mice brains after exposure to THC, maternal immune activation, or both, it was found that densities of CB1 and CB2 receptors had decreased significantly [47], which can lead to risks of altered neuroanatomical developments. Even worse, other studies in mice show that even just a single dose of THC administered during a critical brain development period can decrease transcription levels in the parietal cortex and hippocampus, providing evidence that exposure to THC during a sensitive period of brain development can not only disrupt neurotropic signaling, but also increase oxidative stress and even apoptosis [48]. On the other hand, previous research has shown that exposure to Δ9-THC can alter immune responses in blood cells; however, the mechanisms underlying these changes in gene expression remain limited [49]. All taken together, current literatures suggests that Δ9-THC exposure can pose significant neurodevelopmental and immunological risks, particularly during critical periods of brain development.

Unlike Δ8- and Δ9-THC, there is very limited research on the toxicology of Δ10-THC, including its isomers (*trans-* and *cis-*Δ10-THC) [21]. Δ10-THC isomers are primarily created synthetically or semi-synthetically via isomerization from hemp-derived CBD [21]. At present, most information about Δ10-THC is derived from its structural similarity to Δ8- and Δ9-THC, including its relatively low toxicity [50]. However, current research suggests that Δ10-THC is generally less potent at CB1 receptors [21] **(**Table 1**)**, likely producing milder psychoactive effects. Recent effects reported from cases related to Δ10-THC include mild nervous system depression, agitation, and more [51].

While all Δ8-, 9-, and 10- share a foundation as positional isomers, their toxicology and effects differ due to structural variations and potency. Δ9-THC remains the most potent isomer with documented therapeutic benefits. Δ8- and Δ10- may exhibit lower potency and toxicity, but Δ8- can raise metabolic concerns at higher doses [29], [50].

## 5 Biological actions of THC & CBD

### 5.1 Anti-inflammatory & inflammatory effects of THC via their receptor interactions

**Fig. 1:**
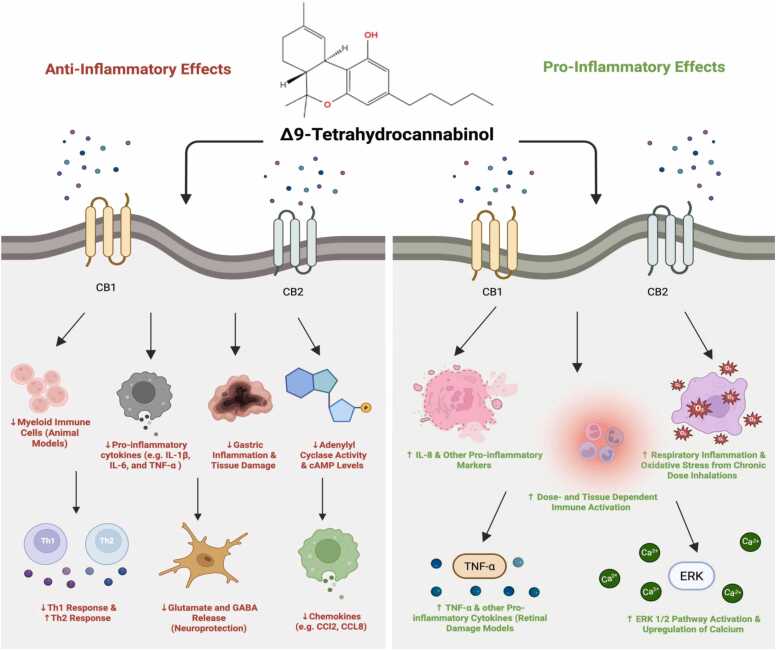
Anti-Inflammatory and Pro-Inflammatory Effects of THC. THC, which primarily acts on CB1 and CB2 receptors, can exhibit several anti-inflammatory properties. THC can suppress T cell cytokines, myeloid immune cell infiltration in animal models, and chemokines [55]. Activation of the CB1 receptor also downregulates GABA and cAMP levels [56]. Meanwhile, activation of the CB2 receptor inhibits adenylyl cyclase activity [56] and reduces modulation of T cell activity by Th-1 and Th-2 cells, thereby decreasing the production of pro-inflammatory cytokines (e.g., IL-1β, IL-6, and TNF-α) [57]. Nonetheless, when THC is inhaled at chronic doses, it may also provoke pro-inflammatory effects, including upregulation of TNF-α and other pro-inflammatory cytokines in retinal damage models and increase respiratory inflammation [58]. Furthermore, when CB2 is activated, it may also increase intracellular calcium levels, leading to activation of the ERK 1/2 pathway and oxidative stress-induced damage in human retinal epithelial cells [59]. This figure was created in Biorender.com.

Interestingly, current literature searches indicate that Δ9-THC does not significantly affect the NF-κB signaling pathway, the central inflammatory regulator, in BV-2 microglial cells, unlike CBD [52]. Compared to THC isomers, CBD more strongly suppress LPS-induced NF-κB activation, beyond CB1 and CB2 receptor pathways [53]. In fact, mechanistic studies on the effects of Δ8- and Δ10-THC on NF-κB signaling or downstream inflammatory pathways are very limited, and NF-κB specific data for these newer isomers remain largely unavailable. However, THC does generally exhibit several anti-inflammatory properties [54]. In animal models specifically, THC has demonstrated a reduction in myeloid immune cell infiltration and suppression of chemokines and T cell cytokines, which thereby reduce inflammatory processes [55] (Fig. 1). This is shown to protect against different forms of gastric inflammation and tissue damage [55]. As previously discussed, THC primarily acts on CB1 and CB2 receptors, which are both G-protein coupled receptors [56], [114], [115]. The binding of ligands to CB1 and CB2 induces a conformational change in the receptors, initiating different downstream cellular processes [56]. Different molecular dynamic studies have shown that ligand binding to CB1 and CB2 receptors occurs via lateral insertion from the lipid bilayer [56]. Activation of CB1 is strongly linked to GABAergic and glutamergic cells, suppressing the release of glutamate and gamma-aminobutyric acid (GABA). It has also been discovered that activation of the CB1 receptor increases the activity of potassium and calcium ion channels and inhibits adenylate cyclase, leading to decreased cyclic adenosine monophosphate (cAMP) levels [56] (Fig. 1). On the other hand, CB2 receptor activation inhibits adenylyl cyclase through the Gi/Go subunits, affecting mature and neoteric tissues [56]. Activation of CB2 receptors by THC has been shown to decrease the production of pro-inflammatory cytokines such as IL-1β, IL-6, and TNF-α [57]. Furthermore, CB2 receptor activation also reduces modulation of T cell activities via inhibiting Th-1 and promoting Th-2 cells [57]. All taken together, the release of glutamate and GABA could suggest THC’s potential anti-inflammatory benefits for neuroprotection and mood regulation (Fig. 1).

However, THC’s diverse impact on inflammation and receptor signaling can also vary by dose, exposure duration, tissue type, and the condition. Other contradictory studies have suggested that just THC alone does not generally reduce pro-inflammatory cytokines by a significant amount but can increase TNF-α in retinal damage mouse models [58] (Fig. 1). In fact, when inhaled at higher chronic doses, it may also provoke pro-inflammatory responses through different respiratory irritation mechanisms [58]. Furthermore, other CB2 mechanistic research shows that when CB2 is activated via an agonist, an increase in intracellular calcium levels and oxidative stress-induced damage occur in human retinal epithelial cells [59] (Fig. 1). Although the study did not directly investigate THC, the study suggests that THC, as a partial CB2 agonist, could potentially elicit similar receptor-mediated inflammatory responses under certain cellular conditions. Yet, such an effect appears to be context-dependent and requires further research. Such complexity and variety in receptor targets may help account for the diverse pharmacological impacts of THC observed across different tissues.

### 5.2 Anti-inflammatory & inflammatory mechanisms of CBD via NF-κB pathways

**Fig. 2:**
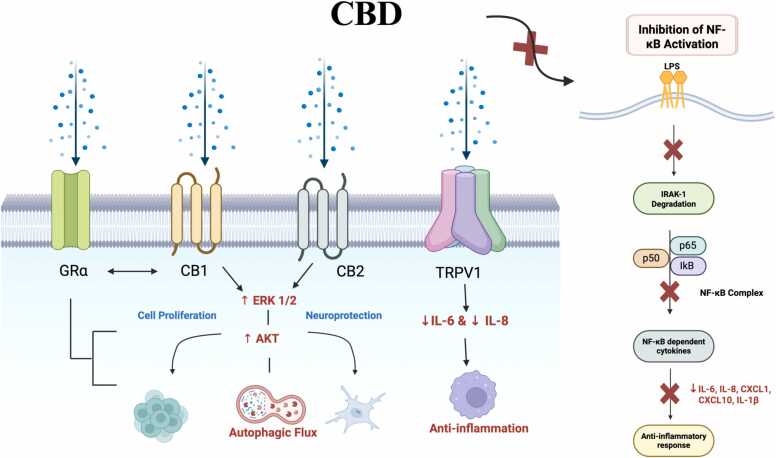
Potential Anti-inflammatory Pathways via CB1, CB2, and TRPV1 Receptors and NF-κB Activation. The major non-psychotomimetic phytocannabinoid, CBD, exhibits neuroprotective, antioxidant, antiemetic, and anticarcinogenic properties through various mechanisms. CBD interacts with CB1 and CB2 receptors, activating ERK 1/2 and AKT suppression, ultimately mediating autophagic flux in the cell. AKT suppression activation is also associated with anti-inflammatory effects, including cell proliferation, neurological function, neuroprotection, and immune modulation. TRPV1 activation leads to a reduction in IL-6 and IL-8, promoting anti-inflammatory effects. Interestingly, CBD also reduces NF-κB-dependent pathways by inhibiting IkB and IRAK-1 degradation and reducing p65 phosphorylation, thereby limiting pro-inflammatory cytokine expressions. CBD also reduces the activation of LPS and STAT1, thereby inhibiting pro-inflammatory mediators. As shown above, other anti-inflammatory properties are also caused by reduced cytokines and chemokines. CB1/CB2 receptors can interact with the glucocorticoid receptor (GR) alpha to override the effect of steroids, rendering steroids ineffective. This figure was created in Biorender.com.

CBD, the major non-psychotomimetic phytocannabinoid, shares neuroprotective, antioxidant, antiemetic, and anticarcinogenic qualities with THC and has been shown to counteract some of THC’s adverse effects, such as sedation and intoxication [17], [19], [45]. Like THC, CBD interacts with CB1, CB2, and TRPV1 (Transient Receptor Potential Channel Vanilloid Type 1) receptor antagonists, which play a role in its anti-inflammatory effects. Similarly, it may interact with other receptors like GRα, to override the effect of steroids via CB1/CB2-GRα interaction, thereby rendering steroids ineffective [27], [56] (Fig. 2).

Recent studies have discovered that CBD mediates an autophagic flux through ERK1/2 and AKT suppression activation, which is associated with neuroprotection, cell proliferation, and survival [54], [116] (Fig. 2). Remarkably, CBD also reduces NF-κB dependent pathways, a central inflammatory regulator, and upregulates STAT3, a transcription factor that induces anti-inflammatory processes [53], [116]. Mechanistically, recent studies suggest that CBD, not THC, modulates NF-κB regulations by inhibiting IkB and IRAK-1 degradation and NF-κB p65 phosphorylation [52] (Fig. 2). However, both CBD and THC reduce the activation of the LPS, STAT1 transcription factor, a significant unit in another pro-inflammatory activity, IFNβ [52]. Other studies have also further elucidated CBD’s potent anti-inflammatory effects in human microglial cells, due to the reduction of various cytokines and chemokines, including IL-6, IL-8, CXCL1, CXCL10, and IL-1β [53], [116] (Fig. 2). These findings can further highlight potential therapeutic applications involving CBD in neuroinflammation.

Similar to THC, CBD may also exhibit pro-inflammatory effects under certain conditions, such as specific doses or cell types, showing a more nuanced picture. Research on rheumatoid arthritis synovial fibroblasts specifically indicate that CBD can increase calcium levels through TRPA1 activation and mitochondrial pore opening, thereby indirectly modulating inflammation by targeting pathogenic fibroblasts [60]. High levels of calcium signaling activate inflammatory pathways in other cells [60]. Furthermore, other findings show that CBD can suppress IL-1β in human monocytes but has mixed effects on IL-6. Interestingly, IL-6 is suppressed in some receptor-activated monocytes but increased in others [58]. Regardless, these context-dependent actions underscore the need for further research of CBD’s dual role in immune regulation, which may also influence its therapeutic potential and safety profile. All taken together, these receptor interactions and mechanistic pathways can offer a biological framework for interpreting the beneficial and harmful health outcomes of CBD and THC isomers.

## 6 Current cannabinoid regulations

As discussed previously, the 2018 Farm Act Bill detailed significant changes in U.S. agricultural and regulatory policies, particularly in hemp production. Once signed into law, the 2018 Farm Bill legalized the general commercial cultivation of hemp by amending the Controlled Substances Act (CSA) to exclude “hemp” from the definition of “marijuana” [61]. This distinction was made by classifying hemp as cannabis (*Cannabis sativa* L.) and derivatives of cannabis with no more than 0.3 % of the psychoactive compound, Δ9-THC. Other cannabis plants or derived products with greater than 0.3 % Δ9-THC are defined as “marijuana” under the legislation and continue to be federally illegal. Additionally, the U.S. 2018 Farm Bill removed hemp from being considered a Schedule I substance by removing the phrase “[THC] in hemp” from THC listings in Schedule I [61].

The repercussions of this framework include the expansion of hemp cultivation, which is detailed in Section 10113 of the 2018 Farm Bill. Section 10113 provides a framework for state and tribal authorities to submit their plans for monitoring and regulating hemp production to the U.S. Department of Agriculture (USDA). If a state does not submit a plan or if it is not approved, then the USDA will implement its plan [61]. Additionally, the legality of the non-psychotropic cannabidiol (CBD) products was clarified, an issue that many legal experts describe as a highly misinterpreted matter in the cannabis industry. The Bill specifically legalizes any cannabinoid derived from hemp plants, thus classifying any marijuana-derived CBD as an illegal Schedule I substance [62].

Other current federal guidelines remain fragmented, particularly regulations concerning novel cannabinoids like Δ8-THC and Δ10-THC. Inadvertently, the specificity of the legal threshold for a substance to be below 0.3 % Δ9-THC has facilitated the rise of novel cannabinoids not explicitly addressed by federal law. Examples of these psychoactive THC isomers currently sold as “legal hemp derivatives” include Δ8-THC, Δ10-THC, THC-O-acetate derivatives, hexahydrocannabinol (HHC), cannabinol (CBN), tetrahydrocannabiphorol (THCP), and tetrahydrocannabivarin (THCV) [39].

This federal “loophole” arises from the vague terminology used to define hemp. By specifying a THC threshold, other potentially psychoactive hemp-derived cannabinoids that do not have the chemical structure of THC, like CBN, are inadvertently legalized [63]. Although Δ8-THC and Δ9-THC both naturally occur in cannabis, Δ8- and Δ9- can be synthesized through a series of chemical reactions from CBD, derived from either hemp or marijuana. Despite Δ9-THC levels being federally restricted, the legislation, alongside most state laws, does not provide regulation of hemp-derived THC isomers, like Δ8-THC. Consequently, a regulatory loophole now exists, where a lack of regulation of these THC isomers equates to no federal marketing or safety regulations of such products [64], though state-licensed dispensaries are established. Other hemp-derived THC isomers are misleadingly marketed as “diet weed” or “marijuana light,” leaving a false impression of safety [63].

The health consequences of this inconsistent regulation have already been noted. According to LoParco et al., Δ8-THC is easily accessible online, less expensive than Δ9-THC, and is marketed in ways that appeal more towards children and teenagers. Also, a lack of safety regulations on the production of such substances has resulted in contaminated products and inconsistent effects, as well as calls to emergency services due to accidental Δ9-THC exposure among minors [65]. Already, poison control centers across the US are encountering incidents of adults mistaking Δ8-THC products for CBD products and minors consuming Δ8-THC gummies believing them to be candy [66].

Another rising concern with the proliferation of novel cannabinoids is the potential for Δ9-THC and other THC isomers as a reaction by-product. A study conducted by the US Cannabis Council found that in a sample of products labeled as Δ8-THC, byproduct Δ9-THC concentrations were, on average, 10 times above the 0.3 % limit. A lack of regulation and inaccurate labeling of such products has also revealed invalid certificates of product analysis for byproducts since the techniques utilized failed to distinguish specific isomers like iso-THCBF (isotetrahydrocannabifuran) from Δ8-THC. An additional study commissioned by CBD Oracle corroborated these differences in results, similar to the previously mentioned study, but in a different sample of products marketed with Δ8-THC. In this group, 76 % of products contained a Δ9-THC level beyond 0.3 %, and most samples failed to report reaction side products, heavy metals, or pesticides [14]. There is a cause for concern for marketing regulation beyond Δ8-THC products as well, since other novel cannabinoids like THCP and THC-O-acetate derivatives require more complex reactions for synthesis, potentially resulting in more undisclosed and undiscovered side products [14]. Further, the regulatory loophole has led to the proliferation of hemp-derived intoxicating cannabinoids like Δ8-THC and Δ10-THC, and other analogs in their various combinations, along with the combinations of CBD/CBN/CBG and Δ9-THC with different flavors in the market.

To address this legal flaw, the Drug Enforcement Administration (DEA) released a statement to clarify that Schedule I controlled substances still include synthetically derived tetrahydrocannabinol. However, there has yet to be evidence of enforcement by the FDA. Also, there is no guarantee that judicial authorities will find Δ8- to be a “synthetically derived tetrahydrocannabinol” since it is already naturally occurring in the cannabis plant in small amounts [67]. More recently, the FDA released warning letters to five companies for misleading packaging of their food products containing Δ8-THC that were introduced into the marketplace in violation of the Federal Food, Drug, and Cosmetic Act [68]. While the federal government’s reaction towards novel cannabinoids has been limited, at least 21 States have blocked the sale of Δ8-THC, with other states enforcing more restrictive measures against Δ8-THC products in recent years [67]. Yet, it remains unclear whether existing regulations apply to State-licensed dispensaries regarding these products.

Federal actions also apply outside of Δ8-THC products. In 2023, the U.S. Drug Enforcement Agency (DEA) clarified that THC-O acetate derivatives are classified as Schedule I drugs due to their synthetic and non-naturally occurring nature, thus not falling under the definition of hemp-derived [63]. THC-O acetate derivatives are almost three times as potent as traditional THC [69]. Similarly, in April 2024, the U.S. DEA determined that HHC does not naturally occur in the Cannabis sativa L. plant, thus not falling under the definition of hemp. Although earlier studies have indeed reported detecting small amounts of HHC in cannabis using gas chromatography-mass spectrometry, indicating that HHC can occur naturally, typically in trace amounts [70]. Despite this, concentrations found in cannabis are generally too low to be considered for commercial extraction [70]. Hence, nearly all HHC products available on the market are produced synthetically or semi-synthetically. Due to HHC’s predominant synthetic origin, it marks the most recent novel cannabinoid to be excluded from the Bill’s legalization [71]. This exclusion is now officialized by H.R. 8467, the Farm Bill’s extension for the 2025 fiscal year. The U.S. Congress enacted this extension on December 21, 2024, and signed into law in December 2024 [72]. Additionally, this bill intends to redefine the current definition of hemp to be based on the “total tetrahydrocannabinol” concentration as opposed to Δ9-THC [73]. Despite several state attempts at closing the regulatory loophole, the ambiguity in the current framework with novel cannabinoids still poses serious health concerns.

## 7 Health effects of CBD/THC

### 7.1 Beneficial health effects of CBD/THC

Mechanistic pathways, such as NF-κB pathways and CB1/CB2 receptor activation, as discussed earlier in Section 5, can contribute to many downstream clinical outcomes. For instance, the therapeutic potentials of THC and CBD, when used appropriately under medical supervision, are also notable. Currently, several cannabinoid-based medications have received FDA approval including Syndros, Cesamet, Marinol, and Epidiolex [74] (Table 3). Furthermore, in addition to the various health effects of different forms of THC, as shown in (Table 4), the beneficial effects of CBD and THC can also depend on dosage, often resulting in different biological outcomes. Currently, although only Δ9-THC and CBD are well-studied forms of cannabinoids—responsible for intoxicated states and a range of therapeutic properties, respectively—cannabis has historically been valued for its pain-relieving, anti-inflammatory, and calming effects [75], [76]. Recent literature findings indicate that substantial clinical trial evidence supports the use of high oral doses of CBD, specifically 10–50 mg/kg, for treating childhood epilepsies [75]. In a study assessing the efficacy and tolerability of oral CBD doses through clinical trials, researchers found that the therapeutic benefits of CBD become apparent only when doses are ≥ 300 mg [75]. Furthermore, while there appears to be no increase in adverse effects for doses between 60 and 400 mg/day, evidence suggests that at doses of 300–400 mg/day, reduced anxiety and anti-addiction effects occur in drug-dependent individuals [75].

**Table 3:** Current FDA-approved cannabinoid-based therapeutics.

**Drug**	**Active Cannabinoid Compound**	**Regulatory Status of Prescription Drug & Clinical Indications**
**Syndros**	Dronabinol, also known as synthetic Δ9-THC [74].	FDA-approved [74] Used for weight loss for patients with AIDS and treatment of anorexia; chemotherapy-induced nausea [74].
**Cesamet**	Nabilone, a synthetically derived cannabinoid structurally similar to THC [74].	FDA-approved [74]. Chemotherapy-induced nausea for cancer patients [74].
**Marinol**	Dronabinol, also known as synthetic Δ9-THC [74].	FDA-approved [74] Used for weight loss for patients with AIDS and treatment of anorexia; chemotherapy-induced nausea [74].
**Epidiolex**	Purified, plant-derived form of CBD [74].	FDA-approved [74]. Used for treatment of seizures for patients with Dravet syndrome, Lennox-Gastaut Syndrome, and tuberous sclerosis [74].

**Table 4:** Different forms of THC and their associated health effects.

**Form of THC**	**Common Health Effects**
**Δ8-THC**	• Euphoria, relaxation, pain relief, impaired memory, anxiety, dry mouth, increased appetite [16], [18] • Hallucinations, sedation, tremor, anxiety, dizziness, confusion, visual distortions, and loss of consciousness [77]
**Δ9-THC**	• May lead to euphoria, relaxation, anxiety, dizziness, dry mouth [16], [18]
**Δ10-THC**	• May lead to mild euphoria, increased energy, clarity, mild anxiety, dry mouth, dizziness, sedation, and confusion [16], [18]

Other studies have also shown cannabinoids to have significant effects on managing chronic pain, seizures, convulsions, nausea, peripheral neuropathy, inhibiting cancer cell growth and inflammatory bowel disorders, and lowering blood glucose levels [76]. As previously discussed, CBD can mitigate some of the adverse psychotropic effects of THC by acting as a negative allosteric modulator against CB1 [22] (Table 1), giving rise to its anti-inflammatory properties, such as anti-seizures [78]. Other chronically high levels of THC lower the efficiency of parts of the brain that control planning and cognitive tasks [79]. Despite all the potential benefits under medical supervision, when cannabis overuse occurs, the risk of psychotic symptoms can also be readily apparent in later life [76]. At present, the explicit ratio of THC: CBD for medical therapeutic purposes has not yet been identified in the literature search and requires further research.

### 7.2 Harmful health effects of CBD/THC

While some findings highlight the therapeutic potentials of CBD and THC under medical supervision, unregulated and high-dose, chronic exposures to these substances can lead to a wide range of adverse health outcomes. Due to the lack of strict regulatory control over cannabinoids, adverse health risks will continue to emerge. This concern is particularly relevant when the thermal degradation of flavored e-cigarettes can generate oxidants, heavy metals, and volatile organic compounds [80]. Not only have CECs raised significant concerns within the medical community through public health and secondary exposure concerns, as previously discussed, but the misuse of THC and CBD can also lead to various complications affecting brain tissue and inducing pulmonary effects [3], [81]. Specifically, Chan et al. found that lung samples from EVALI patients exposed to excessive inhalation of THC showed abnormal lipid macrophages and symptoms such as shortness of breath, dyspnea, nausea, chest pain, vomiting, fever, diarrhea, and weight loss [82]. Similarly, studies involving mouse models and *in vitro* experiments with human cells have demonstrated that cannabinoid vaping products can alter immune response and result in severe lung damage. Specifically, in tissues exposed to higher levels of CBD aerosol inhalation, myeloperoxidase activity and neutrophil elastase levels were significantly elevated compared to regular nicotine aerosol, resulting in more pronounced inflammatory changes and lung injury caused by oxidative stress [83].

CEC and THC can also cause complications affecting biochemical and molecular processes in brain tissue. Many past neuroimaging studies reveal that morphological abnormalities are associated with the amount of cannabis exposure. High levels of CB1 receptors are present in the cerebellum, neocortex, and temporal lobe, but they are expressed at lower levels in other brain regions [12]. Within the temporal lobe, studies have shown that elevated CB1 levels coincide with reduced hippocampal size in 14 young adults with a history of heavy CEC use (5.8 joints/day) for over 6 months [12], [13]. Other research indicates that individuals dependent on cannabis exhibit significant abnormalities in hippocampal volume, where long-term users are more likely to have a smaller amygdala and hippocampal volume [12].

Nevertheless, numerous findings suggest that when CB1 levels are densely expressed in the temporal lobe, hippocampal volume can be significantly altered, potentially leading to long-term functional memory deficits and brain damage [13]. In addition to volume abnormalities, studies have discovered that frequent cannabis exposure is particularly detrimental to the adolescent brain, where the right hippocampus shows negative correlations with the number of cannabis joints smoked. This finding indicates that lower hippocampal volumes and impaired neural connectivity are associated with greater cannabis use, especially among adolescents [12], [13], [84].

Aside from the previously mentioned concerns, the existing loopholes in federal cannabinoid regulations, along with colorful packaging and flavorful marketing strategies from manufacturers in vaping products and fruity flavors, can serve as a gateway and significantly increase underage consumption [1]. Numerous pre-clinical and epidemiological data have indicated that excessive use of CECs may lead to various addictive behaviors over time. Furthermore, it is linked to higher risks of anxiety, depression, and lung cancer [84]. One study found that exposing juvenile rodents to cannabinoids decreased dopamine reactivity in the brain’s reward region [84], [85]. If early exposure to CBD indeed diminishes dopamine levels during adolescence, this likely explains the consistent pattern of heightened vulnerability to addiction and substance abuse later in life [84].

### 7.3 Cannabinoid hyperemesis syndrome link to THC

Aside from the previously mentioned concerns and benefits of CBD/THC, the present loopholes in federal cannabinoid regulations, along with colorful and flavorful marketing strategies, can serve as a gateway to increase consumption among young adults significantly, further leading to harmful health risks. For example, studies have found that chronic and habitual cannabis usage, especially high-potency cannabis that is THC-rich, has been linked with cannabinoid hyperemesis syndrome (CHS), another harmful consequence of CBD and THC [5], [8]. For example, in one study, an extensive survey was conducted of all CHS patients, 89 % highlighted that they used 4 g of THC-predominant cannabis daily on average, with THC concentrations higher than 15 % [8]. Hallmarks of CHS are often characterized by continued episodes of severe nausea, cyclic vomiting, and abdominal pain [6]. Because of such overlapping symptoms with other common diseases, CHS can often be overlooked and underdiagnosed [6]. Even worse, hallmarks of CHS are well known to be resistant to standard antiemetics [9]. Hence, patients often exhibit compulsive patterns of bathing and showering for simple symptomatic relief [86]. As cannabis use increases, especially with the current regulatory loopholes, user numbers can rise over time, as well as the number of CHS cases.

Presently, although the complete pathophysiology of CHS has not been fully understood, several literatures have proposed strong links between CHS and different factors, including CB1 and CB2 receptors, TRPV1, and inflammation control via the release of specific pro-inflammatory mediators (e.g., interleukins). One possible mechanism explains that THC can act as a partial agonist at CB1 and CB2 receptors. Specifically, at lower doses of THC, activation of CB1 can suppress nausea and vomiting signals [87], whereas at chronic high levels of THC exposure, CB1 receptors can be downregulated, and THC can accumulate in the cerebral fat tissues, triggering vomiting during the stress of fasting [87], [88]. Furthermore, as previously mentioned, CB2 receptors play a significant role in inflammation control; hence, chronic cannabinoid exposure can dysregulate the gut and immune function, further contributing to CHS symptoms [87]. Other genomic studies have identified mutations in the TRPV1 gene amongst patients with CHS, which encodes the capsaicin receptor involved in sensing heat and pain. When TRPV1 is activated by capsaicin, it leads to an emetic response (vomiting) [89].

Interestingly, recent studies have also suggested that certain genetic polymorphisms, such as variants in the dopamine D2 receptor (DRD2), cytochrome P450 enzymes (CYP2C9), ATP-binding cassette transporter (ABCA1), and catechol-O-methyltransferase (COMT), have been linked to increased CHS susceptibility [8]. Particularly, mutations in the DRD2 gene are a great indication of drug addiction beyond cannabis, also susceptible to chronic pain, psychosis, and anxiety [8]. On the other hand, the enzyme CYP2C9 helps facilitate THC metabolism by converting THC into 11-hydroxy-THC (active metabolite) and then into inactive 11-nor-9-carboxy-THC [89]. However, a mutation that causes hypoactivity of CYP2C9 can have toxic effects due to slowing down the rate of THC metabolism as the rate of catabolism of 11-hydroxy-THC becomes impaired [89]. Finally, other literature has found that variants in the ABCA1 transporter can increase the risk of dementia, abnormal brain protein depositions, and coronary artery diseases [8]. COMT facilitates dopamine breakdown; hence, when mutations of COMT are suspected, patients may be linked to poor antidepressant responses, psychosis, anxiety, and addiction [89]. Above all, as previously mentioned, the complete pathophysiology of CHS is limited and not fully understood; further research is needed to define the complete mechanisms underlying CHS, especially if user numbers are expected to increase over the years due to the regulatory loophole.

At present, complete cessation of cannabis use appears to be the most effective treatment for CHS since conventional antiemetics are ineffective in relieving symptoms [90]. Recent literature also suggests various promising treatments for CHS, including capsaicin, haloperidol, benzodiazepines, and olanzapine [9]. As previously discussed in relation to capsaicin’s link to TRPV1, treatments like capsaicin creams are reported to help relieve some patients with CHS [9]. Otherwise, benzodiazepines and haloperidol have been reported to be effective, as supported by many trials and case reports. For instance, in one case study, four patients with CHS who had been referred to the emergency department failed all standard therapy for CHS; however, after receiving haloperidol, they all remarkably showed improvement in symptoms and were discharged 8 h after [86]. The mechanism behind haloperidol draws from its role as an antagonist at D2 receptors in the central nervous system. High concentrations of D2 cause the drug’s antiemetic properties for CHS [86]. Recent animal studies have also highlighted complex interactions between CB1 and dopamine [91], which may explain haloperidol’s antiemetic properties as well. On the other hand, benzodiazepine primarily enhances the action of GABA at the GABA-A receptor, leading to a reduction in anxiety, nausea, and vomiting [9]. Likewise, olanzapine, an antipsychotic drug, helps block neurotransmitter receptors such as serotonin and dopamine, leading to antiemetic effects as well [9]. Despite current studies highlighting various promising treatment options, the current scientific evidence for pharmacologic treatment is still limited. This underscores the importance of awareness and health risks associated with cannabinoid consumption, and the need for more clinical trials to define optimal treatment.

## 8 Biomarkers of toxicity

In addition to the positive and negative health effects of CBD and THC, recent literature has also highlighted a range of biomarkers that signal toxic responses to exposure. These biomarkers can serve as indicators of exposure, reflect underlying pathological processes, and identify CEC users at risk of respiratory complications. Many findings show that biomarkers of toxicity for CBD/THC include elevated liver enzymes: alanine aminotransferase (ALT) and aspartate aminotransferase (AST), neurological biomarkers, and endocrine markers. In a clinical study conducted by Florian et al., 201 healthy participants were recruited and subjected to a randomized, double-blind, placebo-controlled trial investigating the effects of CBD dose (2.5 mg/kg/d, twice a day) on endocrine hormones and liver enzymes [92]. After 4 weeks, 8 of 151 participants (5.6 %) had liver enzymes of ALT or AST 3 times the upper limit, while 50 placebo participants showed no concerning results. Additionally, increased eosinophil counts were observed in 7 of 8 participants with elevated ALT levels [92]. ALT and AST play a crucial role in detecting liver cell injury, inflammation, and toxicity [93], which suggests that CBD exposure can result in immune-related hepatic responses, further underscoring the need for better CBD regulations.

Other in vitro animal cases also show that CBD-related toxicities include central nervous system inhibition, liver damage, toxic thyroid hormone levels, embryo-fetal mortality, hypotension, developmental toxicity, and alterations to the male reproductive system with higher doses of CBD than normal human pharmacotherapies [92], [94]. Other than hepatic abnormalities as previously mentioned, human clinical studies for epilepsy have reported CBD to be associated with symptoms like vomiting, fatigue, and somnolence [94]. Finally, in recent studies using fluorescence microscopy, it was discovered that CBD can significantly increase levels of reactive oxygen species (ROS) and calcium, and decrease bound NADPH fluorescence, suggesting risks for oxidative stress and inhibition of the electron transport chain, respectively [95], [96]. All taken together, biomarkers for CBD, such as elevation of liver enzymes, eosinophilia, oxidative stress, and mitochondrial dysfunction, reflect the systemic toxicity, neurotoxicity, and hepatotoxicity associated with CBD exposures.

Moreover, there are also biomarkers of toxicity of cannabis vaping-associated injuries. For instance, one study by McGraw et al. identified a potential noninvasive biomarker for EVALI: plasma phosphatidylethanolamines [97]. In the study, plasma samples with LC-MS/MS revealed over 500 molecular features, demonstrating that phosphatidylethanolamine levels were significantly lower in both EVALI users and e-cigarette users compared to the healthy group [97]. Since phosphatidylethanolamines are classified as a type of plasmalogen in human cells and are the third most abundant phospholipid in pulmonary surfactants, they play a crucial role in homeostasis by maintaining surfactant equilibrium and reducing surface tension [97], [98]. Hence, phosphatidylethanolamines could serve as a viable biomarker for other surfactant-mediated diseases and respiratory environmental exposure, as many specific alterations can be observed in conditions such as ATP-binding cassette transporter A3 deficiency (ABCA3) and pulmonary fibrosis [98].

In addition to phosphatidylethanolamine, various noninvasive systemic biomarkers related to e-cigarette-induced lung injuries, including THC metabolites, oxidative stress markers, lipid mediators, and inflammation indicators, have also been identified in numerous pilot studies [99]. In one study, researchers collected and compared plasma and urine samples from EVALI patients and non-vape users, utilizing Luminex-based assays along with ELISA/EIA biomarkers for analysis. The results demonstrated that EVALI subjects commonly exhibit higher levels of the THC metabolite (11-nor-9-carboxy-Δ9-THC) in their plasma samples compared to non-vape users [99]. Other mediators, such as resolving D1 (EvD1) and prostaglandin E2 (PGE2), were found to be significantly lower in EVALI samples. Elevated levels of 8-hydroxy-2’-deoxyguanosine (8-OHdG) and 8-isoprostane were identified in EVALI urine samples, representing oxidative DNA damage biomarkers and oxidative stress markers, respectively [99]. Lastly, several pro-inflammatory biomarkers, including GM-CSF, TNF-α, and MIP-1β, showed decreased levels in affected plasma samples, which suggests a dysregulated inflammatory response due to e-cigarette use [99]. Overall, these biomarkers of toxicity can serve as effective indicators for earlier diagnosis of cannabinoid-associated respiratory complications and for guiding clinical intervention and treatments for CBD/THC.

## 9 Current public health concerns and secondary exposures to CBD/THC/Nicotine

Given the toxicity of cannabinoids, cannabis vaping (along with nicotine vaping) can pose a significant public health concern if its prevalence is high. Unfortunately, we are witnessing an increase in cannabis vaping among adults, particularly young adults (ages 18 – 24). For adults overall, the rate of past-30-day cannabis vaping doubled from 1 % in 2017–2 % in 2019 [100]. Among young adults, the increase was even more pronounced (from 1.2 % to 3.9 %) [100]. Another study analyzing trends among adolescents found a six-fold rise in the prevalence of past-30-day cannabis vaping, increasing from 1.6 % in 2013–8.4 % in 2020 [2]. This significant rise among adolescents and young adults may be attributed to a lower perceived risk and the misconception that cannabis vaping is a safer alternative to other forms of substance use [101]. The relatively recent emergence of cannabis vaping raises serious public health concerns, especially for youth, due to the limited understanding of its long-term health effects on developing lungs, insufficient regulations, and the prevalence of more potent cannabinoid forms in e-liquids. These issues are compounded by the high rates of use among young people. Additionally, cannabis vaping has seen a more considerable increase among males than females, as well as among heterosexuals compared to homosexuals. Meanwhile, no significant differences were observed among ethnic, educational, and socioeconomic groups [100].

Similar to cannabis smoke, second-hand exposure to cannabis aerosols can also pose a public health risk [102]. Particles are emitted by e-cigarette users at levels comparable to those of traditional cigarette users, suggesting that while cannabis vaping produces fewer particulates than cannabis smoking, it may still adversely affect air quality around the user [103], [104]. The decline in air quality can increase the risk of bronchial problems among young adults (the demographic with the highest rate of cannabis vaping), particularly in those who lack awareness of how to avoid such exposures [105]. One recent controlled study involving simulated cannabis vaping aerosols observed that particle emissions from cannabis vaping greatly decrease indoor air quality while also exposing pollutants to other non-vape users [106]. Degraded indoor air quality was evident as Tang et al. found that second-hand particle concentrations (PN) ranged from 0.7 × 106–13 × 106 per cm^3^ and mass concentrations ranged from 65 to 1191 μg/m^3^ [106]. These peak levels indicate that emissions from cannabis vape exposure can expose non-users to hazardous and high levels of toxicants, especially when emitted concentrations can be comparable to severe air pollution events [106]. Furthermore, second-hand exposure may also worsen existing airway conditions, including asthma [107]. Although there is no direct data regarding cannabinoid content in second-hand cannabis e-cigarette emissions, pharmacokinetic studies on cannabinoid absorption via inhalation indicate an efficiency of 10–35 % for THC and 31 % for CBD [108]. This suggests that the user might not absorb a significant amount of cannabinoids in the aerosol, allowing them to be exhaled as second-hand exposure, potentially exhibiting the toxicities discussed previously. Such second-hand exposure to cannabinoids can also raise public health concerns.

## 10 Regulatory policies on cannabis and cannabis research

As previously discussed, cannabis availability has significantly increased over the last two decades. It is now accessible for medical purposes in most states, and in nearly half of the states, adults are permitted to purchase it for recreational use [109]. The rapid changes in cannabis availability and policy have outpaced our understanding and scientific research of its effects, despite the current literature and studies. Given the swiftly evolving landscape of cannabis, cannabis policy is crucial for guiding informed public health decisions. According to the National Survey on Drug Use and Health, it was reported that in 2022, 15.1 % of individuals were currently using cannabis, and young adult (age 18–25) cannabis use rose from 19.5 % to 23.3 % between 2012 and 2019 [110]. This increase can be attributed to vape packaging, vibrant colors, and flavors designed to attract young adolescents [80]. However, various cannabis and vaping industries have continuously introduced an array of products with very high concentrations of THC [111]. Following an independent consensus study conducted by the National Academies of Sciences, Engineering, and Medicine, the NIH’s comprehensive roadmap for cannabis research report currently outlines the public health consequences of changes in cannabis policy, different regulatory frameworks for research and marketing approaches in other states, and enumerates recommendations for research to be conducted at the federal, state, and tribal levels to better inform policy [112].

Additionally, the roadmap discussed evaluations of various health programs that promote health and social equity, the impacts of the high potency of synthetic cannabinoid products, the therapeutic benefits of cannabis for health, and the interactions with prescription drugs. Other recommendations aim for the development of better tests to detect cannabis impairment, stricter and improved surveillance of cannabis cultivation and product sales, and broader adoption of a standard 5 mg THC unit in research studies [112]. Despite this new roadmap, public awareness and health initiatives focused on prevention are still necessary to prevent common misconceptions about cannabis vaping being safer than traditional smoking methods.

### 10.1 Evolving risks and urge for regulatory oversight on the current cannabis landscape

All taken together, although cannabis has been used since ancient times and traditionally cultivated in natural forms with low concentrations of active compounds like Δ9-THC, the current cannabis market has undergone substantial changes. Modern strains now contain much higher and potentially hazardous levels of THC, which may contribute to adverse health effects such as EVALI and CHS, as previously discussed. Alongside, the diversity of THC isomers and minor compounds has increased, many of which remain poorly understood in terms of pharmacological and toxicological profiles. Moreover, the rise of different synthetic cannabinoids implies that recreational users are likely subjected to substances that are not naturally present in plants. These synthetic compounds often have greater drug potency and unpredictable effects, and their unregulated status raises further concerns about unknown health risks. This lack of oversight increases the risk that recreational users may encounter substances that are bacteriologically and chemically harmful. Hence, regulatory frameworks must be strictly strengthened to ensure that cannabis products are professionally handled and tested, overall promoting public health, and minimizing harm to users.

### 10.2 Toxicity and regulatory sciences

As discussed earlier, there are numerous issues with the current regulatory framework for cannabis vaping, including the lack of regulation of Δ8- and Δ10-THC, as well as inconsistencies in law enforcement across administrations and states. Therefore, robust regulatory updates should be implemented to address the public health concerns associated with these regulatory loopholes, particularly given their significant adverse impact on youth and young adults [65].

One way to address the issue is to broaden the scope of regulation beyond Δ9-THC in hemp derivatives. The current 0.3 % THC limit for hemp products should instead focus on the concentration of total cannabinoids that exhibit similar effects to Δ9-THC, including Δ8-, Δ9-, Δ10-THC, and others. Furthermore, regulatory agencies must establish a cohesive regulatory framework for the prompt and consistent enforcement of regulations rather than the fragmented approach currently in place [67]. Given the rising popularity of cannabis vaping among young people [2], [100], educating them about the risks associated with such products is also crucial. This education can be facilitated by regulating the messages presented on product packaging, which has demonstrated some educational effectiveness [113]. Furthermore, additional research on the properties of various cannabinoid forms is needed to provide a stronger scientific foundation and better inform the development of innovative regulatory strategies.

In addition, although FDA-approved CBD products have documented therapeutic benefits, such as treating seizures in patients with Dravet or Lennox-Gastaut syndromes [74], these benefits must be weighed against emerging evidence of hepatotoxicity and further adverse effects at higher doses. Evidence from *in vitro* and preclinical studies further suggests that CBD aerosols can induce more pronounced lung injuries and pulmonary inflammation compared to nicotine aerosols alone [83], underscoring great concern for inhaled and vaping formulations containing unknown levels of CBD. Such toxicity findings can be utilized to inform regulatory measures and guide limits on CBD and THC concentrations in vaping products or disposables and a stricter requirement for clear labeling that disclose accurate doses, thereby aligning product oversight with health risks and reducing misuse of CBD in largely unregulated markets. Although this review primarily focuses on inhaled and vaping formulations with CBD/THC, the rise of high-dose oral CBD products such as drug infused gummies, is also of safety and regulatory concern equally.

## 11 Conclusion

The emergence and growing popularity of cannabinoids and tetrahydrocannabinol, including Δ8-, 9-, and 10-THC, present significant health risks and public health concerns. While cannabinoids and Δ9-THC do demonstrate some therapeutic medical potential according to medical reports, their widespread availability, especially regarding products like Δ8- and Δ10-THC, remains poorly regulated. Lack of consistent regulation enables misuse and raises significant public health concerns. Due to the existing loopholes in the regulation regarding hemp products, Δ8-THC and other THC forms are readily available through online platforms, where vibrant packaging and appealing marketing strategies target adolescents and young adults. Additionally, counterfeit e-cigarettes introduce a range of toxic substances, including solvent-derived hydrocarbons, silicon-linked chemicals, and vitamin E acetate. These compounds can contribute to oxidative stress and inflammatory responses, leading to acute respiratory syndromes like EVALI, as suggested by earlier studies. Beyond the toxicological risks, the pharmacological effects of THC and CBD through CB1 and CB2 receptors and NF-κB pathways reveal complex roles in neuroprotection, inflammation, and immune modulation. It has divergent pro- and anti-inflammatory properties based on dose and susceptibility factors. Although some literature suggests that young adults have the highest prevalence of cannabis vaping, this review emphasizes the critical need for regulatory reforms by mandating a more consistent cannabinoid regulation across states, more accurate labeling of cannabinoid content in e-cigarette products on the market, and expanding the scope of limited regulation beyond Δ9-THC. Establishing proactive measures is essential to safeguard public health and mitigate health risks by reducing the rise of novel cannabinoids and exposure to harmful unlabeled chemical additives. Public awareness and public health initiatives focusing on prevention are necessary to counteract common misconceptions about cannabis vaping being ‘safer’ than traditional methods. Ultimately, this review underlines the urgency for further research and tighter regulatory frameworks to mitigate health risks among youth associated with cannabis vaping.

## CRediT authorship contribution statement

**Irfan Rahman:** Writing – review & editing, Writing – original draft, Resources, Methodology, Investigation, Funding acquisition. **Felix Effah:** Writing – original draft, Methodology. **Karen Lin:** Writing – original draft, Methodology. **Parth Suharu:** Writing – original draft, Methodology. **Rhea Raghu:** Writing – original draft, Methodology. **Yehao Sun:** Writing – original draft, Methodology.

## Consent to participate, and consent to publish declarations

Not applicable.

## Ethics

Not Applicable.

## Funding

None.

## Declaration of Competing Interest

The authors declare that they have no known competing financial interests or personal relationships that could have appeared to influence the work reported in this paper.

## Data Availability

No data was used for the research described in the article.
